# 双特异性T细胞衔接器治疗急性淋巴细胞白血病中国专家共识（2024年版）

**DOI:** 10.3760/cma.j.cn121090-20240528-00194

**Published:** 2024-07

**Authors:** 中国临床肿瘤学会白血病专家委员会 中国临床肿瘤学会淋巴瘤专家委员会 中华医学会血液学分会 中国医师协会血液科医师分会

**Keywords:** 白血病，淋巴细胞，急性, 双特异性T细胞衔接器, 免疫治疗, Leukemia, lymphoblastic, acute, Bispecific T cell engager, Immunotherapies

## Abstract

急性淋巴细胞白血病（acute lymphoblastic leukemia, ALL）是最常见的急性白血病之一，发病急、进展快，化疗和移植的规范化应用已极大改善患者预后，但仍存在未满足的治疗需求。近年以双特异性T细胞衔接器（Bispecific T cell Engager，BiTE）为代表的免疫治疗发展迅速，为ALL治疗提供了更多选择，同时也对临床诊疗管理提出了更高的要求。专家组基于国内外最新循证医学证据、相关指南和临床实践，对2022年版《双特异性T细胞衔接器治疗急性淋巴细胞白血病指导原则》进行更新，制定了该版中国专家共识。

## Abstract (en)

Acute lymphoblastic leukemia (ALL) is one of the most common acute leukemias, with rapid onset and progression. The standardized application of chemotherapy and transplantation have improved the prognosis of patients, while the unmet therapeutic needs still exist. Recently novel immunotherapies including Bispecific T cell Engager develop rapidly, offering more options for ALL treatment and also demanding higher requirements for clinical diagnosis and treatment management. Based on the evidence of domestic and international medical evidence and clinical experience, the expert panel updated Chinese consensus for the Bispeific T cell Engager in the treatment of B-cell acute lymphoblastic leukemia (2022) and formulated this edition of the Chinese expert consensus.

前体B细胞急性淋巴细胞白血病（Precursor B-cell acute lymphoblastic leukemia, BCP-ALL）是一种进展迅速的白血病，表现为骨髓、外周血和其他器官存在过多B淋巴细胞母细胞。根据2019年全球疾病负担报告，全球新发153 320例ALL患者，且新发病例数逐年增加[1]。成人新诊断ALL患者经多药化疗治疗之后有较高的完全缓解（CR）率，长期生存率30％～50％，但复发风险高，复发或难治性（R/R）患者的中位总生存（OS）期仅为2～6个月[2]。

贝林妥欧单抗作为第一个在全球范围内被批准的双特异性T细胞衔接器（Bispecific T cell Engager，BiTE），能够与B细胞表面的CD19和T细胞表面的CD3结合，从而介导T细胞与肿瘤细胞间形成免疫突触、上调细胞黏附分子、产生细胞水解蛋白、释放炎性细胞因子并促进T细胞增殖，定向裂解杀伤CD19阳性肿瘤细胞[3]。贝林妥欧单抗在中国获批治疗成人和儿童R/R CD19阳性的BCP-ALL，并被FDA批准治疗第1次完全缓解（CR_1_）或第2次完全缓解（CR_2_）伴微小残留病（MRD）≥0.1％的成人及儿童BCP-ALL，新近又获批一线巩固适应证（不论MRD状态）[4]。专家组基于国内外最新循证医学证据、相关指南和临床实践，对2022年版《双特异性T细胞衔接器治疗急性淋巴细胞白血病指导原则》[5]进行更新，从而为临床医师应用贝林妥欧单抗治疗提供指导。

一、BiTE技术平台及贝林妥欧单抗药学特点

BiTE抗体结构是由两个单链可变区通过灵活的肽链连接而成，一侧结合域可识别肿瘤表达抗原（如CD19、CD33、BCMA等），另一侧通常特异性识别CD3，即T细胞受体复合物的成分之一，从而衔接T细胞和肿瘤靶细胞，引起T细胞活化并杀伤肿瘤靶细胞，因此是针对各种肿瘤的疗法，并且接受再治疗仍可能有效。与其他双抗相比，BiTE具有相对分子质量小、肿瘤穿透作用强、单链可变区之间的连接柔韧性更好等特点[6]。

贝林妥欧单抗的药代动力学特征呈线性，主要分布在血管内，平均半衰期为2.11 h，平均全身清除率估算为3.11 L/h，因此需连续静脉给药28 d以维持有效稳态浓度[7]。儿童患者（<18岁）的清除率随体表面积（body surface area, BSA）增加而增加，成人患者未观察到明显相关性，因此体重<45 kg患者采用基于BSA的给药方案，而≥45 kg成人患者给予固定剂量的给药方案[7]–[8]。轻度或中度肾功能不全的患者亦无需调整剂量。

药效学反应表现为T细胞活化和再分布、外周血B细胞减少和一过性的细胞因子升高。连续输注贝林妥欧单抗后，外周CD3^+^ T细胞呈现“再分布”现象：1～2 d内下降至极低水平，随后在7～14 d内迅速恢复至基线，持续输注2～3周后大部分患者的T细胞数量扩增至基线的2倍以上，提示持续输注贝林妥欧单抗对T细胞有扩增作用[8]–[10]。

贝林妥欧单抗输注后2 d内可观察到IL-6、IL-10和TNF-γ等细胞因子升高幅度最大，在24～48 h内返回至基线。后续治疗周期中发生细胞因子水平升高的情况较少，因此贝林妥欧单抗的免疫治疗相关不良反应多发生在第一治疗周期的7～10 d内[8]–[10]。

二、贝林妥欧单抗治疗成人ALL临床研究

1. Ph阴性新诊断成人B-ALL：多中心Ⅲ期随机对照研究（ECOG-ACRIN E1910）[11]–[12]纳入224例诱导治疗后达到MRD<0.01％的新诊断Ph^−^ B-ALL成人患者，分别接受贝林妥欧单抗4个周期联合化疗或化疗巩固，随后进入POMP方案（6-巯基嘌呤、长春新碱、甲氨蝶呤和泼尼松）维持治疗。中位随访43个月，80％试验组患者接受≥2个周期贝林妥欧单抗治疗，57％接受了4个周期治疗。贝林妥欧单抗联合化疗组的中位OS期未达到，较化疗组71.4个月显著延长（*HR*＝0.42，95％ *CI* 0.24～0.75，双边*P*＝0.003）。

GMALL研究组多中心Ⅱ期临床研究（EWALL-BOLD）[13]使用减低剂量的化疗序贯贝林妥欧单抗诱导巩固（共计4个周期）治疗50例老年新诊断Ph^−^ B-ALL患者，中位年龄66岁，其中40％合并糖尿病、心血管疾病等并发症。低剂量化疗及1个周期贝林妥欧单抗诱导后CR/CRu率为85％，CR患者中分子学完全缓解率为82％（MRD阴性，灵敏度0.01％），均较1个周期化疗有进一步提升（76％和18％）。中位随访757 d，1年OS率为80％，3年OS率为67％。仅3例患者在CR_1_接受了异基因造血干细胞移植（allo-HSCT）。对比化疗历史数据［CR率78％，分子学完全缓解率55％（*P*＝0.006），3年OS率49％（*P*＝0.08）］，提示贝林妥欧单抗替代部分化疗治疗初治老年患者可提升缓解率和改善生存结局，且安全性良好。

2. Ph阳性新诊断成人B-ALL：GIMEMA协作组Ⅱ期临床研究[14]–[15]纳入63例新诊断的Ph^+^ B-ALL患者，中位年龄54岁，诱导治疗为激素预治疗序贯达沙替尼联合泼尼松，巩固治疗为达沙替尼联合贝林妥欧单抗2～5个周期。诱导治疗后血液学CR率98％，分子学缓解率仅29％，贝林妥欧单抗2个周期巩固治疗后分子学缓解率60％，4周期后提升至81％。中位随访53个月，无病生存（DFS）率75.8％，OS率80.7％。研究中半数患者仅使用TKI作为维持治疗，未进行系统化疗或移植，证实了一线无化疗方案为患者带来持久的高质量缓解及生存获益。其中BCR::ABL p210阳性患者的分子学缓解率和DFS率与p190阳性患者差异无统计学意义，提示贝林妥欧单抗可能克服BCR::ABL p210的不良预后。

MD Anderson癌症中心一项Ⅱ期临床研究[16]–[17]采用贝林妥欧单抗最多5个周期联合Ponatinib治疗62例新诊断Ph^+^ ALL患者。40例可评估患者中，血液学缓解率98％。55例可评估分子学反应患者中，治疗1个周期后完全分子学缓解（CMR）率67％，治疗期间最佳CMR率84％。其中47例二代测序（NGS）检测患者MRD阴性（灵敏度为10^−6^）率为91％。2年无事件生存（EFS）率为77％，2年OS率为89％，仅1例患者在CR_1_接受了HSCT。再次证实无化疗方案能够为一线Ph^+^ ALL患者带来较高CMR率和长生存。

3. R/R成人B-ALL：TOWER研究[2]已证实贝林妥欧单抗在R/R B-ALL成人患者中的治疗地位，血液学缓解率（CR/CRh/CRi）为44％，中位OS期为7.7个月，相比于传统化疗组缓解率显著提高，生存期延长（4个月，*HR*＝0.71，*P*＝0.01），首次复发时贝林妥欧单抗的治疗获益相较化疗更加显著，中位OS期分别为11.1个月和5.3个月（*HR*＝0.6）[2],[18]。

一项法国Ⅱ期临床研究探索了贝林妥欧单抗联合VANDA强化疗方案（依托泊苷、阿糖胞苷、米托蒽醌、地塞米松和门冬酰胺酶）治疗17例R/R B-ALL患者的初步疗效[19]。CR率为82％，65％患者接受了HSCT。中位随访17个月，1年OS率64.7％，1年无白血病生存（LFS）率58.8％。移植患者的1年LFS为90.9％。VANDA-BLI方案开始至中性粒细胞恢复的中位时间仅15 d。提示贝林妥欧单抗无论单药还是联合化疗，均可为复发患者带来高缓解率，为移植创造条件。

此外，贝林妥欧单抗联合PD-1单抗、CTLA-4单抗或Bcl-2抑制剂等其他免疫靶向药物的研究也在早期探索中，疗效和安全性有待进一步验证。

三、贝林妥欧单抗治疗儿童ALL临床研究

1. 新诊断儿童B-ALL：ALL在儿童肿瘤中占75％～80％，5年生存率约89％，但高危患儿仍易复发，婴儿白血病患者生存率改善有限[20]。

澳大利亚白血病和淋巴瘤工作组在ALL06研究中使用基于BFM（Berlin-Frankfurt-Munster）方案治疗新诊断的15～40岁青少年和年轻成人（AYA）患者，化疗巩固后（第79天）MRD阴性率60％，3年OS率74.9％，且第79天MRD阴性率与生存预后显著相关。因此，研究者在ALL09 SuBliME研究[21]将贝林妥欧单抗连续输注28 d替代部分巩固化疗，评估对第79天MRD阴性率的影响。早期数据显示，在可评估的48例患者中，贝林妥欧单抗巩固后MRD阴性率显著高于ALL06研究（70.8％对60％，*P*＝0.037）。另一项Ⅱ期试验针对14岁及以上新诊断Ph^−^B-ALL患儿，强化疗诱导治疗后予贝林妥欧单抗巩固治疗4个周期，后续POMP与贝林妥欧单抗交替共维持治疗15个周期[22]。中位随访37个月，预估3年无复发生存（RFS）率为73％，贝林妥欧单抗巩固带来的长期生存令人鼓舞。

一项国际多中心Ⅱ期研究纳入30例伴KMT2A重排的新诊断婴儿白血病患者，在Interfant-06化疗方案基础上加入1个周期贝林妥欧单抗治疗[23]，93％患者达到MRD阴性（无法检测到MRD，16例）或低水平MRD（<5 × 10^−4^，12例）。中位随访26.3个月，2年DFS率为81.6％，2年OS率为93.3％，均高于Interfant-06方案历史数据（49.4％和65.8％）。无贝林妥欧单抗相关不良事件导致的停药或死亡。

正在进行中的Ⅲ期随机对照研究（AIEOP-BFM ALL 2017）[24]比较2个周期贝林妥欧单抗替代部分高强度巩固化疗或传统化疗巩固治疗高危B-ALL患儿。初步安全数据显示，贝林妥欧单抗组的特别关注不良事件和严重不良事件的发生率均低于化疗组（10.3％对22.8％，*P*<0.001；0对5.2％，*P*<0.001）。

2. R/R儿童B-ALL：allo-HSCT是R/R儿童B-ALL的重要治疗手段，但中高危患儿如早期复发者常因化疗相关不良反应（严重感染等）无法接受移植，以及再诱导治疗后无法获得MRD阴性而降低移植预后。

多中心随机对照Ⅲ期研究（215研究）[25]针对诱导化疗及两个周期巩固治疗达到形态学CR（M1骨髓，原始细胞<5％）或M2骨髓（5％≤原始细胞<25％）的高危首次复发患儿，予贝林妥欧单抗对比化疗作为第3周期巩固治疗，贝林妥欧单抗组显示出更高MRD缓解率（90％对54％）。在治疗第29天MRD<10^−4^患者中，贝林妥欧单抗组MRD不可测得/可测得但不可定量的比例更高（*P*＝0.037）[26]，且桥接allo-HSCT比例更高（88.9％对70.4％）。中位随访44个月，贝林妥欧单抗组生存结局显著优于化疗组（4年EFS率：59％对27％，*P*<0.001；4年OS率：77％对49％，*P*＝0.002），同时安全性更好（3级及以上不良事件发生率分别为61％和83％）[27]。

多中心Ⅲ期临床研究（COG AALL-1331研究）[28]纳入208例1～30岁中高危首次复发患儿，诱导化疗后随机予贝林妥欧单抗或化疗2个周期巩固治疗。贝林妥欧单抗组的MRD阴性率更高（75％对32％，*P*<0.001），成功移植率也更高（70％对43％，*P*<0.001）。中位随访2.9年，贝林妥欧单抗显著延长患儿的生存结局（2年DFS率：54.4％对39.0％，*P*＝0.03；2年OS率：71.3％对58.4％，*P*＝0.02）。此外，该研究（COG AALL-1331研究）低危队列[29]也显示出更长生存趋势（估计4年DFS率：61.2％对48.2％，*P*＝0.15；4年OS率：91.6％对83.3％，*P*＝0.096），骨髓复发伴或不伴髓外复发的亚组生存获益显著（4年DFS率：74.0％对51.8％，*P*＝0.016；4年OS率：96.6％对84.4％，*P*＝0.013）。贝林妥欧单抗的安全性显著优于化疗，包括≥3级发热伴粒缺、感染、贫血和黏膜炎等。

四、真实世界经验

美国多中心真实世界研究回顾了239例使用贝林妥欧单抗治疗的成人B-ALL患者[30]，其中227例为R/R患者，12例为MRD阳性患者，26％患者既往接受过≥3线治疗。贝林妥欧单抗治疗中位2个周期后，R/R患者的CR/CRi率为65％（MRD阴性率为47％），中位RFS期为32个月，中位OS期为12.7个月；MRD阳性患者中，MRD阴性率75％，随访2年中位RFS期未达到，中位OS期为34.7个月。≥3级细胞因子释放综合征（CRS）、神经毒性和肝脏毒性的发生率分别为3％、7％和10％。此外，249例NEUF研究组真实世界数据显示[31]，Ph^+^和Ph^−^ R/R ALL患者经贝林妥欧单抗治疗2个周期后CR/CRh/CRi率分别为41％和51％，MRD完全缓解率分别为67％和83％，中位OS期分别为16.3个月和12.2个月；Ph^+^和Ph^−^ MRD阳性患者1个周期贝林妥欧治疗后MRD完全缓解率分别为64％和93％，中位随访18.5个月，中位OS期未达到。

我国单中心回顾了96例使用贝林妥欧单抗治疗的成人B-ALL患者[32]，包括53例新诊断患者和43例R/R患者。贝林妥欧单抗中位用药周期数为1（1～5）个。可评估的新诊断患者中，MRD完全缓解率为93.6％。R/R患者中有28例使用贝林妥欧单抗再诱导缓解，CR/CRi率为50％。中位随访205 d，新诊断和R/R患者的1年EFS率分别为90.8％和55.1％。研究中仅1例患者发生≥3级神经毒性，3例发生≥3级CRS事件。这些结果都验证了贝林妥欧单抗在真实世界中应用的疗效和安全性。

儿童同情用药项目回顾性收集了2014–2017年间通过项目接受贝林妥欧单抗治疗的113例儿童B-ALL患者数据[33]。其中72例为R/R患儿，2个周期贝林妥欧单抗治疗后CR率为53％，其中MRD阴性率为83％，中位RFS期为5.4个月，中位OS期为8.2个月；36例可评估的MRD阳性患儿中，75％获得治疗反应，59％桥接HSCT，中位随访12.5个月，中位DFS期为13.6个月，中位OS期未达到。该研究证实了贝林妥欧单抗在儿童患者中的真实世界疗效。

五、贝林妥欧单抗治疗B-ALL的临床应用推荐

基于以上贝林妥欧单抗在成人及儿童患者中的研究和真实世界数据，各国指南针对贝林妥欧单抗在B-ALL中的应用做了相关推荐的更新（表1）[20],[34]–[35]。

**表1:** 贝林妥欧单抗治疗成人B-ALL的临床应用推荐

患者	指南	治疗阶段	治疗推荐
新诊断的Ph^−^ B-ALL	CSCO	标危组，MRD持续阴性高危组，或MRD阳性	有条件者贝林妥欧单抗联合化疗巩固（Ⅰ级推荐） <65岁，allo-HSCT或贝林妥欧单抗清除残留治疗后桥接allo-HSCT（Ⅰ级推荐） ≥65岁，多药联合化疗方案巩固治疗后进入维持治疗或贝林妥欧单抗清除残留治疗后进入维持治疗（Ⅰ级推荐）
	NCCN	巩固（不论MRD状态）	诱导治疗获得CR后不论MRD状态，推荐贝林妥欧单抗联合或不联合化疗治疗（其他推荐）
	ESMO	巩固（MRD阴性）	诱导治疗后，MRD阴性，使用贝林妥欧单抗巩固可提升患者的治疗结局（Ⅱ，A）
		巩固（MRD持续阳性）	诱导或巩固化疗后，MRD持续阳性，使用贝林妥欧单抗巩固可提升MRD反应和治疗结局（Ⅰ，A）
新诊断的Ph^+^ B-ALL	CSCO	巩固治疗	MRD持续阴性，≥65岁，有条件者贝林妥欧单抗联合TKI巩固治疗后进入维持治疗（Ⅰ级推荐） MRD阳性，<65岁，贝林妥欧单抗清除残留治疗后桥接allo-HSCT（Ⅰ级推荐） MRD阳性，≥65岁，有条件者贝林妥欧单抗联合TKI巩固治疗后进入维持治疗（Ⅰ级推荐），贝林妥欧单抗治疗后进入维持阶段（Ⅱ级推荐）
	NCCN	一线治疗	推荐贝林妥欧单抗联合或不联合TKI（其他推荐）
	ESMO	一线治疗	使用达沙替尼或ponatinib联合贝林妥欧单抗可提升分子学缓解率以及生存结局（Ⅲ，A）
R/R B-ALL	CSCO	再诱导	贝林妥欧单抗（Ⅰ级推荐）
	NCCN	再诱导	推荐贝林妥欧单抗治疗（优先推荐，类别1）
	ESMO	再诱导	贝林妥欧单抗单药治疗R/R B-ALL患者的疗效优于标准多药化疗（Ⅰ，A） 建议对于高肿瘤负荷的患者在应用贝林妥欧单抗之前进行减瘤处理（Ⅳ，B） 应用免疫检查点抑制剂联合贝林妥欧单抗的策略很有前景（Ⅲ，B）

**注** CSCO：中国临床肿瘤学会；NCCN：美国国立综合癌症网络；ESMO：欧洲肿瘤内科学会；MRD：微小残留病；B-ALL：急性B淋巴细胞白血病；R/R：复发/难治；TKI：酪氨酸激酶抑制剂；allo-HSCT：异基因造血干细胞移植；CR：完全缓解

1. 成人B-ALL：专家组治疗推荐：①对于新诊断的Ph阴性B-ALL患者，推荐患者诱导治疗结束达到CR后不论MRD状态，可使用贝林妥欧单抗巩固治疗，以延长患者生存；对于新诊断的Ph阳性B-ALL患者，贝林妥欧单抗联合TKI诱导缓解可作为推荐，诱导结束达到CR后不论MRD状态，可使用贝林妥欧单抗联合或不联合TKI巩固治疗。②对于R/R患者，越早用贝林妥欧单抗生存获益越多，贝林妥欧单抗可作为首次挽救治疗选择推荐，以达到更快更深的缓解，后续桥接allo-HSCT可获得更长生存，对于高肿瘤负荷的患者在应用贝林妥欧单抗之前进行减瘤处理。③对于经诱导治疗获得CR伴MRD阳性的患者，不论CR_1_或CR_2_，贝林妥欧单抗均可推荐用于MRD清除治疗，以获得更持久的缓解和延长生存。

2. 儿童B-ALL：2024年第5版NCCN指南[36]指出，新诊断Ph阴性患儿在诱导治疗后获得CR伴MRD阳性，可推荐贝林妥欧单抗治疗，后续桥接allo-HSCT；Ph阳性高危患儿（诱导治疗未达CR、巩固治疗结束仍存在MRD），可推荐贝林妥欧单抗治疗，后续桥接allo-HSCT。新诊断婴儿白血病伴随KMT2A重排患者，可推荐使用Interfant化疗联合或不联合贝林妥欧单抗治疗，随后继续Interfant强化化疗巩固方案；不伴KMT2A重排患者，诱导后MRD阳性推荐贝林妥欧单抗治疗，后续桥接allo-HSCT。首次复发的B-ALL患儿，诱导治疗后达到CR，不论MRD状态如何，均可使用贝林妥欧单抗治疗，后续考虑桥接allo-HSCT；移植后首次复发患儿，及多次复发或难治患儿，可使用贝林妥欧单抗再诱导治疗。

专家组治疗推荐：新诊断的高危、化疗不耐受、婴儿白血病患者可使用贝林妥欧单抗联合化疗诱导缓解和巩固治疗；R/R患者，越早使用贝林妥欧单抗获益越多，推荐贝林妥欧单抗用于首次复发的挽救治疗和早期复发、诱导后MRD阳性即中高危患者的巩固治疗，后续桥接allo-HSCT可获得更长生存。

六、临床应用管理

1. 早期用药及注意事项：在开始贝林妥欧单抗治疗前对患者的肿瘤负荷进行评估以避免严重CRS的发生，包括骨髓中白血病原始细胞比例≥50％或外周血原始细胞计数>15 000/µl。针对高肿瘤负荷的患者，在开始贝林妥欧单抗治疗前需给予地塞米松进行预先治疗（不超过24 mg/d），并在每个治疗周期第1次给药前1 h、升高剂量前以及中断治疗4 h后重启输注时，预先给予20 mg地塞米松[2],[37]。

2. 输注管理：建议至少第1周期的前9 d以及第2周期的前2 d住院治疗。所有后续周期的启动和重启治疗（例如治疗中断4 h或以上），建议在医疗专业人员监督下或住院进行[37]。前期住院的目的是监测和管理毒性，如输注反应、CRS、肿瘤溶解综合征和神经毒性，一旦患者建立耐受、临床稳定，可以在医护团队随访下转至院外继续治疗。加拿大肿瘤护理协会（CANO/ACIO）建议对于MRD清除患者，第1周期前3 d和第2周期前2 d住院治疗[38]。

院内启动贝林妥欧单抗输注的时间建议在工作日白天，以保障有足够的医护资源应对可能发生的不良事件。由住院输注过渡至门诊或居家输注时，需与患者及家属沟通准确的输液袋更换时间，并确保每次于相同时间更换，确保更换时医疗机构有足够人手提供服务[38]。院外输注可以减少院内感染风险，提高医疗资源利用。建议对进行院外输注的患者进行教育，记录居家日志，告知可能发生的不良反应以及如何进行日常护理。关于便携式注射泵，告知患者不要擅自改变输液泵上的设置，即使当输液泵发生异常、发出警报音时，也不要擅自操作。如果注射泵发生警报尽快联系医务人员[39]。

3. 免疫相关不良反应管理：BiTE分子在临床应用中值得关注的是CRS及神经毒性[37]。在真实世界研究中，≥3级CRS和神经毒性的发生率分别为3％和7％。CRS发生中位时间为输注开始后2 d，CRS消退中位时间为5 d。大多数CRS是可逆的，在贝林妥欧单抗给药前通过识别高危患者、地塞米松预先用药及逐步增加剂量的给药方式进行预防，CRS发生后通过中断贝林妥欧单抗治疗、根据分级评估给予糖皮质激素和IL-6受体阻滞剂治疗和（或）支持治疗，大多数患者在CRS消退后可继续贝林妥欧单抗的治疗[2],[39]。神经毒性发生中位时间为输注开始第9天，大多数神经系统事件可自行消退[2],[38]。所有≥3级的神经毒性均立即暂停给药并进行体格检查、生命体征监测及安全性相关实验室检查，根据患者情况给予地塞米松治疗，每日剂量至少24 mg，地塞米松减量时应于4 d内完成减量直至停药。支持治疗如癫痫发作的抗癫痫药物应由专科医师判断酌情使用[2],[40]。贝林妥欧单抗的≥3级血液学不良反应发生率明显低于化疗，常见的（≥20％）不良反应包括感染（细菌性和未指明病原体）、发热、头痛、输注相关反应、贫血、发热性中性粒细胞减少症、血小板减少症和中性粒细胞减少症。接受贝林妥欧单抗治疗的儿童患者中的不良反应的类型与复发或难治性BCP-ALL成人患者中观察到的相似。针对不良反应的剂量调整见表2[37]。

**表2:** 发生CRS、神经系统毒性和其他临床相关不良反应时剂量调整

不良反应	分级^a^	体重≥45 kg的患者	体重<45 kg的患者
CRS	3级	中断本品输注 每8 h通过静脉或口服给予地塞米松8 mg，共3 d，然后在4 d内逐步减量 CRS消退后，使用9 µg/d的剂量重启本品治疗，如果CRS未复发，则在7 d后升高剂量至28 µg/d	中断本品输注 每8 h通过静脉或口服给予地塞米松5 mg/m^2^（最高8 mg），共3 d，然后在4 d内逐步减量 CRS消退后，使用5 µg·m^−2^·d^−1^的剂量重启本品治疗，如果CRS未复发，则在7 d后升高剂量至15 µg·m^−2^·d^−1^
	4级	永久终止本品治疗。根据发生3级CRS时的说明给予地塞米松	
神经系统毒性	惊厥	发生超过1次惊厥发作，则永久终止本品治疗	
	3级	暂停本品治疗直至神经系统毒性事件不超过1级（轻度）且持续至少3 d，然后以9 µg/d的剂量重启治疗。如果神经系统毒性事件未复发，则在7 d后升高剂量至28 µg/d。如果9 µg/d剂量下发生不良事件，或者神经系统毒性事件在超过7 d后才消退，则永久终止本品治疗	暂停本品治疗直至神经系统毒性事件不超过1级（轻度）且持续至少3 d，然后使用5 µg·m^−2^·d^−1^的剂量重启治疗。如果神经系统毒性事件未复发，则在7 d后升高剂量至15 µg·m^−2^·d^−1^。如果5 µg·m^−2^·d^−1^剂量下发生神经系统毒性事件，或者神经系统毒性事件在超过7 d后才消退，则永久终止本品治疗
	4级	永久终止本品治疗	
其他临床相关不良反应	3级（感染除外）	暂停本品治疗直至不良事件不超过1级（轻度），然后使用9 µg/d的剂量重启治疗。如果不良事件未复发，则在7 d后升高剂量至28 µg/d。如果不良事件在超过14 d后才消退，则永久终止本品治疗	暂停本品治疗直至不良事件不超过1级（轻度），然后使用5 µg·m^−2^·d^−1^的剂量重启治疗。如果不良事件未复发，则在7 d后升高剂量至15 µg·m^−2^·d^−1^。如果不良事件在超过14 d后才消退，则永久终止本品治疗
	4级	考虑永久终止本品治疗	

**注** ^a^基于不良事件通用术语标准（CTCAE），3级为重度，4级为危及生命。CRS：细胞因子释放综合征

七、总结

贝林妥欧单抗以B淋巴系肿瘤高表达的CD19为靶点，利用自身T细胞对肿瘤的免疫作用，有效杀伤肿瘤细胞并清除MRD，越来越多的循证医学证据显示其在成人及儿童B-ALL治疗中的疗效和耐受性。贝林妥欧单抗与化疗的早期联合，以及与其他新药的联合也在积极探索中。随着临床应用的逐渐规范化，贝林妥欧单抗将使更多BCP-ALL患者获益。
